# Prefrontal activation may predict working-memory training gain in normal aging and mild cognitive impairment

**DOI:** 10.1007/s11682-016-9508-7

**Published:** 2016-02-03

**Authors:** Anouk Vermeij, Roy P. C. Kessels, Linda Heskamp, Esther M. F. Simons, Paul L. J. Dautzenberg, Jurgen A. H. R. Claassen

**Affiliations:** 1 Donders Institute for Brain, Cognition and Behaviour, Radboud University, Nijmegen, The Netherlands; 2 Department of Geriatric Medicine, Radboud University Medical Center, Route 925, P.O. Box 9101, 6500 HB Nijmegen, The Netherlands; 3 Department of Medical Psychology, Radboud University Medical Center, Nijmegen, The Netherlands; 4 Department of Geriatric Medicine, Jeroen Bosch Hospital, ‘s-Hertogenbosch, The Netherlands

**Keywords:** Alzheimer’s disease, Cognitive training, Neuroimaging, Optical imaging, Plasticity, Working memory

## Abstract

Cognitive training has been shown to result in improved behavioral performance in normal aging and mild cognitive impairment (MCI), yet little is known about the neural correlates of cognitive plasticity, or about individual differences in responsiveness to cognitive training. In this study, 21 healthy older adults and 14 patients with MCI received five weeks of adaptive computerized working-memory (WM) training. Before and after training, functional Near-Infrared Spectroscopy (fNIRS) was used to assess the hemodynamic response in left and right prefrontal cortex during performance of a verbal n-back task with varying levels of WM load. After training, healthy older adults demonstrated decreased prefrontal activation at high WM load, which may indicate increased processing efficiency. Although MCI patients showed improved behavioral performance at low WM load after training, no evidence was found for training-related changes in prefrontal activation. Whole-group analyses showed that a relatively strong hemodynamic response at low WM load was related to worse behavioral performance, while a relatively strong hemodynamic response at high WM load was related to higher training gain. Therefore, a ‘youth-like’ prefrontal activation pattern at older age may be associated with better behavioral outcome and cognitive plasticity.

## Introduction

Cognitive training has been suggested as a powerful tool to prevent or reduce cognitive decline in normal aging (Lustig et al. 2009; Brehmer et al. 2014) and mild cognitive impairment (MCI; Li et al. 2011; Gates et al. 2011; Reijnders et al. 2013). MCI refers to a clinical condition which is characterized by cognitive decline that exceeds that of normal aging, in the absence of impaired daily functioning (Petersen et al. 2001). MCI patients are at high risk of developing dementia, but no effective pharmacological treatment exists. Therefore, early cognitive intervention may be an attractive non-pharmacological therapeutic approach. Indeed, studies have shown training-related improvements in behavioral performance in various cognitive domains, but it remains unclear which intervention program is most effective in MCI (for reviews, see Li et al. 2011; Gates et al. 2011; Reijnders et al. 2013). Neuroimaging studies investigating cognitive training-induced changes in brain function are necessary to understand the underlying mechanisms of these behavioral improvements and the extent of adaptive brain responses in normal aging and MCI patients.

Recently, a few neuroimaging studies have provided evidence for adaptive brain responses in MCI patients by showing training-induced increased hippocampal activity (Rosen et al. 2011; Hampstead et al. 2012), increased activity in brain areas that were not recruited before training (Belleville et al. 2011; Hampstead et al. 2011), and increased connectivity between brain regions (Hampstead et al. 2011). This pattern of primarily increased activation after training in MCI differs from the observed pattern in healthy older adults, which is characterized by both decreased and increased recruitment of brain regions (Hosseini et al. 2014; Belleville et al. 2014).

Several models of aging-related compensatory brain activation may help to predict and explain training-induced changes in brain activation in normal aging and MCI. Normal aging is associated with decreased activation of task-specific brain regions and increased activation of prefrontal areas, which has been interpreted as a compensatory mechanism. The HAROLD model states that “under similar circumstances, prefrontal activity during cognitive performance tends to be less lateralized in older adults than in younger adults” (Cabeza 2002, p .85). According to the CRUNCH (Compensation-Related Utilization of Neural Circuits Hypothesis) model, activity in prefrontal regions is upregulated as task load increases, irrespective of age. However, aging-related neural decline in older adults may lead to reduced neural processing efficiency, that is, a reduction of the rate and/or quality of neural processing. In order to compensate for reduced processing efficiency, older adults may need to activate task-specific regions to a larger extent and to recruit additional brain regions already at lower levels of task demand to achieve similar performance levels as younger adults (Reuter-Lorenz and Cappell 2008). In comparison to healthy older adults, MCI patients show a further decrease of activity in task-specific brain regions and a decline of the prefrontal compensatory network. It has been proposed that cognitive training in healthy older adults results in normalization of activation in task-specific regions, such as increased hippocampal activation, and reduced prefrontal compensatory activation. In contrast, cognitive training in MCI patients may lead to recovery of activation in both task-specific regions and the prefrontal compensatory network (Hosseini et al. 2014). The INTERACTIVE model of Belleville et al. (2014) adds to these predictions that the training-induced changes in activation may be dependent on the type and method of training. According to this model, training approaches that are directed at normalization of dysfunctional brain regions, such as repeated practice, may result in decreased activation of specialized areas due to improved processing efficiency. In contrast, training approaches that rely on metacognitive processes, such as learning new strategies or mnemonics, may lead to increased recruitment of compensatory networks. In addition, the model states that the pattern of change following training is also dependent on personal characteristics, such as clinical status, proficiency level and cognitive reserve.

The aim of our study was to gain more insight into adaptive responses of the prefrontal cortex in the aging brain by investigating the effects of one specific form of cognitive training: working-memory (WM) training. According to the theoretical framework of Lövdén et al. (2010), cognitive plasticity at older age is driven by a prolonged mismatch between functional supply and task demands. In this framework, functional supply denotes the range of cognitive flexibility of the individual. Training of knowledge-based strategies is likely to affect the range of cognitive flexibility, whereas cognitive process-based training may improve processing efficiency. Targeting a narrowly defined specific process which plays a central role in cognitive functioning, such as WM, will maximize the duration and magnitude of the supply-demand mismatch and may thus induce plasticity (Lövdén et al. 2010; Barulli and Stern 2013). To maximize the generalization effect of the intervention, we therefore selected an adaptive core WM training procedure for this study.

By means of functional Near-Infrared Spectroscopy (fNIRS), prefrontal activation was established during WM performance in healthy older adults and MCI patients before and after five weeks of WM training. fNIRS is a noninvasive hemodynamic neuroimaging technique, which has been proven to be a reliable method in repeated testing studies (Plichta et al. 2006). In comparison to fMRI, fNIRS offers advantages such as lower costs, portability, lower susceptibility to movement artifacts, and high temporal sampling rate (Ferrari and Quaresima 2012). Before and after training, neuropsychological task performance was assessed as well, which was reported by Vermeij et al. (2015). Brehmer et al. (2011) examined the neural correlates of training gain in healthy older adults who received an adaptive WM training program similar to the one used by the current study. After training, the participants showed decreased activity in frontal, temporal and occipital regions during performance of a high WM load task, indicating increased processing efficiency. Individuals who achieved a large behavioral training gain, showed larger decreases of activity in a widespread network of neocortical areas as well as larger increases of activity in subcortical areas and a middle frontal region. Other studies have also reported increased WM performance (e.g., Borella et al. 2010; Brehmer et al. 2012; Buschkuehl et al. 2008; Li et al. 2008; Richmond et al. 2011; Zinke et al. 2014) and decreased frontoparietal activation after process-specific training in normal aging (Dahlin et al. 2008; Erickson et al. 2007). Training-related improvements in WM performance in MCI have been reported by Carretti et al. (2013) and Vermeij et al. (2015), but studies assessing prefrontal activation in MCI patients after adaptive WM training are lacking (Hosseini et al. 2014).

Another unresolved issue is whether prefrontal activation patterns before training may predict behavioral training gain. Heinzel et al. (2014) found that WM training resulted in decreased frontoparietal activation during performance of a low WM load task in healthy older adults. A ‘youth-like’ brain response pattern at baseline, that is, a low BOLD response at low levels of WM load and a high BOLD response at high levels of WM load, predicted better behavioral training outcome. To date, it is unclear whether brain activation patterns may be predictive of WM training gain in MCI.

Based on the models discussed above, we tested the following hypotheses in this study 1) MCI patients show stronger prefrontal activation at lower levels of WM load than healthy older adults in order to maintain performance; 2) WM training leads to decreased prefrontal activation during performance of a high-demanding WM task in healthy older adults, due to increased processing efficiency, and to increased prefrontal activation during performance of a high-demanding WM task in MCI patients, due to restoration of the prefrontal compensatory network; 3) Prefrontal activation is predictive of behavioral training gain; a relatively small hemodynamic response at low WM load and a relatively large hemodynamic response at high WM load are associated with larger behavioral training gain.

## Methods

### Participants

**Fig. 1:**
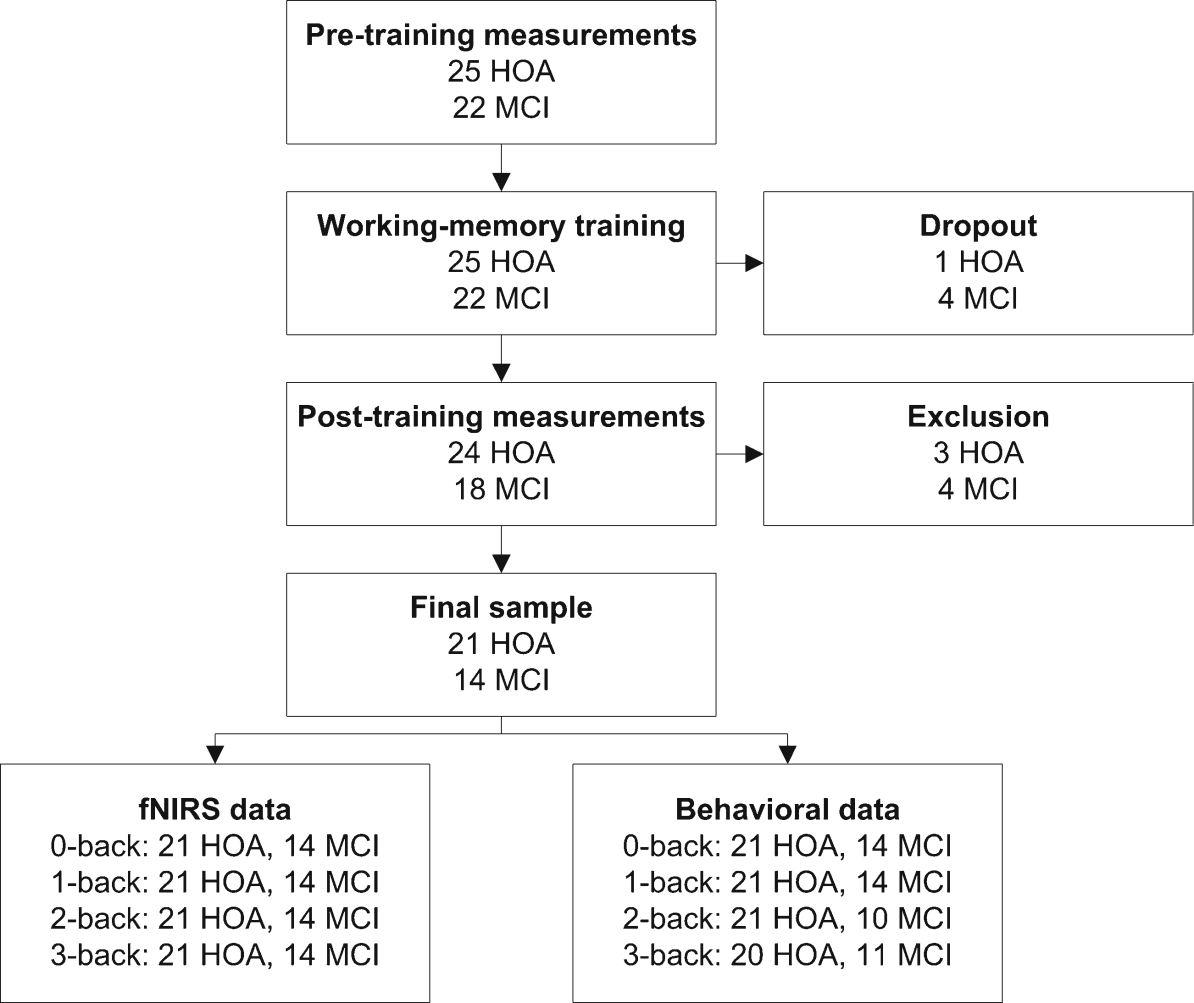
Flowchart of the study. HOA = healthy older adults, MCI = mild cognitive impairment patients

Twenty-five healthy older adults and 22 mild cognitive impairment (MCI) patients were included for participation in this study (Fig. 1). Healthy older adults did not have cognitive deficits and did not have a history of neurological or psychiatric disease. They were recruited from local community centers and an information market organized by the Radboud Alzheimer Center at Nijmegen, The Netherlands. MCI patients were recruited from the memory clinics of the Radboud University Medical Center in Nijmegen, The Netherlands, and the Jeroen Bosch hospital in’s-Hertogenbosch, The Netherlands. MCI patients fulfilled the criteria of Petersen (2004) for amnestic single domain MCI or amnestic multiple-domain MCI. Diagnosis was made using a multidisciplinary approach, including medical examination, medical history, neuropsychological assessment, and/or neuroradiological evidence. At inclusion, overall cognitive functioning was assessed by the Mini Mental State Examination (MMSE; Folstein et al. 1975). Participants were excluded if they scored below the established MMSE cutoff scores (Tombaugh and McIntyre 1992), which was set to <23 for MCI patients and to <27 for healthy older adults.

Figure 1 shows the flowchart of this study. After baseline measurements, five participants withdrew from the study due to lack of time (N = 1), lack of motivation (N = 1), conversion to Alzheimer’s dementia (N = 1), or illness unrelated to the MCI diagnosis (N = 2). In addition, after post-training measurements, seven participants were excluded from data analysis due to a stroke in medical history (N = 1), low fNIRS signal quality (N = 1), or movement artifacts reflected by baseline shifts in the fNIRS signal during task performance, caused by movement of the head or frowning (N = 5). The final study sample consisted of 21 healthy older adults and 14 MCI patients, of whom neuroimaging data were complete and of good quality. With respect to the analyses of the behavioral data, four participants were excluded, because they did not respond to more than 25 % of the trials of one or more working-memory tasks at baseline. One participant was excluded because instructions were not followed correctly during performance of the 2-back task post-training (see Fig. 1).

Table 1 shows the final sample characteristics at baseline. The groups did not differ in years of education, estimated IQ, or body mass index (all *p*-values > .05). Estimated verbal IQ was based on assessment of the Dutch equivalent of the National Adults Reading Test (Schmand et al. 1992). The groups were age-matched at inclusion, but in the final sample, MCI patients were slightly younger than healthy older adults due to drop out (t_(33)_ = 2.03, *p* = .050) (see also Fig. 1). As expected, MCI patients showed a significantly lower MMSE score (t_(15.82)_ = 2.98, *p* = .009). A substantial number of participants received medication, most frequently for hypertension and high cholesterol. Of note, the participants who received psychoactive medication did not meet the criteria for clinical depression or anxiety disorder, and received only a low dose. One may argue that medical conditions, such as type 2 diabetes in four participants, may influence cognitive performance as well. However, in order to be able to generalize our findings to the older population, we aimed to include a representative sample of older adults, maximizing the study’s external validity. In The Netherlands, where this study took place, more than two-thirds of people 65 years and older have two or more chronic conditions for which they receive medication, and 30–45 % of people 65 years and older use five of more types of medication on a daily basis (Lemmens and Weda 2013).

**Table 1:** Sample characteristics at baseline (Mean ± SD)

	Healthy older adults				MCI patients			
Participants	21 persons (13 male, 8 female)				14 persons (10 male, 4 female)			
Age* (years)	69.5	±	5.4	(range 63–81)	66.1	±	3.9	(range 59–72)
Years of education	14.2	±	3.3	(range 9–18)	13.3	±	3.2	(range 9–18)
Estimated IQ	109.8	±	7.2	(range 90–118)	104.3	±	10.9	(range 89–124)
MMSE**	29.2	±	1.0	(range 27–30)	27.1	±	2.4	(range 23–30)
Body mass index	26.1	±	2.9	(range 22.0–32.9)	25.1	±	3.8	(range 19.4–33.6)
Regular cigarette smoker	1 person				1 person			
Alcohol > 2 units/day	2 persons				1 person			
Medication								
Antihypertensives	8 persons				6 persons			
Statins	2 persons				6 persons			
Antiplatelets	4 persons				6 persons			
Antidiabetics	-				4 persons			
Psychoactives (low dose SSRI/benzodiazepines)	1 person				3 persons			

*Note.* *p = .05 **p < .01

### Experimental procedure

#### Overall design

All participants visited the hemodynamics lab of the Radboud University Medical Center in Nijmegen for fNIRS measurements. They performed four conditions of a WM task while their prefrontal activation was established. Following the baseline measurements, they received five weeks of WM training at their own computer at home. Of the final sample, all participants completed all 25 WM training sessions. During the week after completion of the training, the participants returned to the hemodynamics lab, where the fNIRS measurements were repeated.

#### *Assessment before and after WM training*: *N-back tasks*

Before WM training, participants performed four conditions of a verbal n-back task in ascending order: 0-back (control condition), 1-back (low working-memory load), 2-back (medium working-memory load), 3-back (high working-memory load). To get familiar with the tasks, they practiced each condition for one minute and received feedback about their performance. Stimuli were presented in black on a gray background using E-prime 2.0 software (Psychology Software Tools, PA, USA), which also registered the behavioral performance. All conditions consisted of four blocks; each block started with a rest period of 60 s followed by a blank screen for 1 s and a task period of 45 s. During the rest period, a fixation cross was displayed at the center of the screen. The task period consisted of 15 trials, four of which were target trials. In each trial, a letter that was randomly selected from a set of 20 consonants was presented for 500 ms. Interstimulus interval was set to 2500 ms. In the 0-back task, the letter ‘X’ was defined as target. In the other tasks, the target was any letter that was identical to the letter presented n trials back, while the letter ‘X’ no longer appeared. Participants indicated each trial whether the stimulus was a target by pressing the button under the right index finger, or a non-target by pressing the button under the right middle finger (PST Serial Response Box, Psychology Software Tools Inc., PA, USA). After completion of the WM training, participants performed these same n-back tasks.

#### *Cognitive intervention*: *WM training program*

The training procedure and WM training program have extensively been described by Vermeij et al. (2015) in a previous report on this study sample. A commercially available computerized WM training program (Cogmed® QM, Pearson Education, Inc.) was used in this study. After baseline measurements, participants were visited at home during the same week. According to a standardized protocol, instructions about the training procedure were provided by a coach. Participants performed the online training on their own computer. They completed 25 training sessions of approximately 45 min over a period of five weeks. Participants trained five times per week and were not allowed to perform multiple sessions on a single day. The WM training program consisted of twelve verbal and visuospatial WM tasks. Each session, eight WM tasks were selected according to a predetermined order. Task difficulty level was adjusted to the performance level of the individual participant on a trial-to-trial basis by increasing or decreasing the number of items the participant had to remember such that the participant would reach a score of 60 % correct on each task. Such a procedure ensures that the training remains cognitively challenging and that the participant may optimally benefit from the training. Task difficulty level of the new session was based on the achievement levels of the participant during the previous session. Participants were instructed to perform eight tasks in a row, with only short breaks, but they were allowed to split the session if necessary, by taking a longer break after performing four tasks. The participants could keep track of their own achievements. Each week, the coach contacted the participant by telephone to provide support. The coaching conversation was focused on the motivation of the participant. No training strategies, such as ‘chunking of information’, were provided.

### Data acquisition

We measured concentration changes in cortical oxygenated hemoglobin ([O_2_Hb]) and deoxygenated hemoglobin ([HHb]) by means of a continuous-wave NIRS device (Oxymon Mk III, Artinis Medical System, The Netherlands), using light of three wavelengths (765, 857, 859 nm). The NIRS data were sampled at a frequency of 125 Hz. Both increases in [O_2_Hb] and decreases in [HHb] are indicators of cortical activation. To measure prefrontal activation, two pairs of NIRS optodes were bilaterally attached to the forehead and were tightly fixed in a customized headband (Spencer technologies, Seattle, WA). The detection optodes were placed 25–30 mm above the midpoint of the eyebrow of the participant, at approximately FP1 and FP2 according to the international 10–20 electrode system. The emission optodes were laterally placed at approximately F7 and F8. Hence, the cerebral areas under investigation were the left and right superior and middle frontal gyri (Brodmann’s area 10/46; Okamoto et al. 2004). Meta-analysis has shown consistent activation of these areas by various versions of the n-back paradigm (Owen et al., 2005). The interoptode distance between emitter and detector was 50 mm to minimize contamination from the extra-cerebral circulation and maximize signal intensity (van de Ven et al. 2001; van Beek et al. 2012). The age-dependent differential pathlength factor (DPF) accounts for the increased distance traveled by light due to scattering (Duncan et al. 1996). Because no data are available on the actual variation of DPF in adults aged above 50 years, the DPF was set to 6.61, corresponding to age 50, in all participants (Duncan et al. 1996; Claassen et al. 2006).

Complementary physiological measures were used to gain insight into task-evoked systemic processes. Mean arterial pressure (mmHg) was established by means of a photoplethysmography cuff on the index or middle finger of the participant (Finometer, Finapres Medical Systems, The Netherlands) during a 5-min rest period and during n-back performance. A three-lead electrocardiogram was recorded for measurement of the R-R interval in order to determine heart rate (beats per minute).

### Data processing

The fNIRS analyses were performed using MATLAB R2011a (MathWorks, MA, USA). For each n-back task separately, the [O_2_Hb] and [HHb] time series were linearly detrended in order to remove slow drift. A 4th-order low pass Butterworth digital filter with a cut-off frequency of 0.5 Hz was used to filter out high-frequency noise. Next, the data were resampled to 1 Hz. For each task period (that is, four repetitions per n-back task), the [O_2_Hb] and [HHb] time series were baseline corrected by subtracting the mean concentration changes recorded during the last two seconds of the preceding rest period. The selection of a two-second rest period as baseline reference was based on previous block design fNIRS studies (e.g. Müller et al. 2014; Heinzel et al. 2013). The baseline-corrected time series were subsequently averaged over the four repetitions of each task. Finally, for the 0-back, 1-back, 2-back and 3-back task, mean changes of [O_2_Hb] and [HHb] were calculated over the full duration of the task (45 s).

In a subsample of participants, we have compared the use of a two-second rest period with a ten-second rest period as baseline reference. The fNIRS results did not differ between these alternatives. However, some participants had moved their head during the rest periods. The two-second rest period was less likely to have been influenced by movement artifacts and therefore considered to be more reliable compared to the ten-second rest period.

We found a variability in fNIRS response patterns across participants. These patterns were classified into three categories: typical (increase of [O_2_Hb] and decrease of [HHb]), inverse (decrease of [O_2_Hb] and increase of [HHb]), or deviant (increase or decrease of both [O_2_Hb] and [HHb], or no clear response). All response types were included in the analyses. With respect to the left fNIRS channel, the following response patterns were identified: 0-back: typical 45.7 %, inverse 25.7 %, deviant 28.6 %; 1-back: typical 62.9 %, inverse 5.7 %, deviant 31.4 %; 2-back: typical 74.3 %, inverse 0 %, deviant 25.7 %; 3-back: typical 51.4 %, inverse 5.7 %, deviant 42.9 %. For the right channel, we identified the following patterns: 0-back: typical 57.1 %, inverse 17.1 %, deviant 25.7 %; 1-back: typical 77.1 %, inverse 2.9 %, deviant 20.0 %; 2-back: typical 77.1 %, inverse 2.9 %, deviant 20.0 %; 3-back: typical 71.4 %, inverse 0 %, deviant 28.6 %.

### Statistical analysis

#### Behavioral performance

Statistical analysis was performed using IBM SPSS Statistics for Windows version 20.0 (IBM Corp., Armonk, NY, USA). Alpha was set at 0.05 for all analyses. Behavioral performance on the verbal n-back tasks was assessed by number of hits, misses, correct rejections, and false alarms and reaction time. To take the different response types into account, the nonparametric discrimination index (i.e., sensitivity) A′ was calculated by the formula: 0.5 + ((hit rate-false alarm rate) × (1 + hit rate-false alarm rate))/(4 × hit rate × (1-false alarm rate)). A′ is a behavioral performance variable derived from signal detection theory (Grier 1971; Hannay 1988) and ranges from 0.5 (chance level) to 1 (perfect discrimination between targets and non-targets). Furthermore, to take speed/accuracy trade-offs into account and to diminish the influence of strategy effects (McNamara and Scott 2001), composite A′ scores were calculated as 100 × A′/reaction time on targets (ms) × 100. Statistical analyses were performed on these composite A′ scores.

In healthy older adults and MCI patients, the effects of working-memory load and WM training on behavioral performance were established by performing a 2 (group: healthy older adults, MCI patients) × 2 (time: baseline, post-training) × 4 (load: 0-,1-,2-,3-back) repeated measures ANOVA.

#### FNIRS

To investigate the effects of working-memory load and WM training on prefrontal activation, for both hemodynamic measures ([O_2_Hb], [HHb]) and both fNIRS channels (left, right), a 2 (group: healthy older adults, MCI patients) × 2 (time: baseline, post-training) × 4 (load: 0-,1-,2-,3-back) repeated measures ANOVA was performed. Significant main and interaction effects were further analyzed by means of planned contrasts. Effects sizes (η_p_^2^) are reported, which range from 0 to 1. An effect size of η_p_^2^ = 0.01 is considered to be small, η_p_^2^ = 0.06 medium, and η_p_^2^ = 0.14 large (Cohen 1988).

#### Training gain and fNIRS

Training gain (%) was defined as (performance after training–performance before training)/performance before training × 100, where performance represents the composite A′ score averaged over the four n-back tasks. To gain more insight into the relationship between prefrontal activation and behavioral training gain, the whole group was divided into decliners (N = 10; 7 healthy older adults, 3 MCI patients, range training gain = −16.9 % — -1.5 %), low training gainers (N = 10; 8 healthy older adults, 2 MCI patients, range training gain = +0.3 % — +8.8 %), and high training gainers (N = 10; 5 healthy older adults, 5 MCI patients, range training gain = +9.6 % — +26.4 %). The decliners were on average older (*p* = .025) and had fewer years of education (*p* = .037) than the low training gainers. No other group differences were present with respect to age, years of education, estimated IQ or MMSE score (all *p*-values > .05). Independent t-tests were performed to establish group differences in baseline prefrontal activation, and behavioral performance before and after training. Only statistically significant results were reported in section 3.3.1.

For the whole group, Pearson’s (r) partial correlation coefficients (two-tailed, adjusted for age and years of education) were calculated, to establish the relationship between the task-related hemodynamic response at baseline and respectively 1) behavioral performance (composite A′ score) at baseline, 2) behavioral training gain. Correlations were checked and corrected for extreme outliers (> 3 × interquartile range). Only statistically significant results were reported in section 3.3.2.

#### Blood pressure and heart rate

Measures of mean arterial pressure and heart rate were available from 31 participants (19 healthy older adults, 12 MCI patients). Independent t-tests did not show any group effects. The effects of task performance on activation of the sympathetic nervous system were investigated in the total group by means of planned contrasts; n-back performance vs. rest.

## Results

### N-back performance

#### Before training

Table 2 shows the behavioral results in healthy older adults and MCI patients. Performance declined with increasing WM load in both groups (healthy older adults: F_(3,57)_ = 69.93, *p* < .001, η_p_^2^ = .786; MCI patients: F_(3,30)_ = 32.30, *p* < .001, η_p_^2^ = .764). Healthy older adults performed better than MCI patients at 0-back (F_(1,33)_ = 4.45, *p* = .043, η_p_^2^ = .119) and 1-back (F_(1,33)_ = 5.28, *p* = .028, η_p_^2^ = .138).

**Table 2:** Working-memory performance (Mean composite A′ score ± SD) before and after training

Healthy older adults	Pre-training			Post-training		
Composite A′ score						
0-back	17.81	±	2.25	18.40	±	2.90
1-back	15.78	±	2.70	16.18	±	2.23
2-back	11.59	±	2.74	12.09	±	2.57
3-back	9.57	±	2.84	9.60	±	2.89
MCI patients	Pre-training			Post-training		
Composite A′ score						
0-back	16.08	±	2.58	17.04	±	2.72*
1-back	13.41	±	3.38	14.51	±	3.36^~^
2-back	10.94	±	2.55	10.59	±	3.41
3-back	9.05	±	2.80	9.24	±	3.24

*Note.* *p < .05 ^~^trend .05 < *p* < .10 post-training vs. pre-training. Composite A′ scores were calculated as 100 × A′/reaction time on targets (ms) × 100, to take speed-accuracy trade-offs into account. A′ represents a nonparametric discrimination index which takes different response types into account and which was calculated by the formula: 0.5 + ((hit rate-false alarm rate) × (1 + hit rate-false alarm rate))/(4 × hit rate × (1-false alarm rate)

#### After training

In both groups, similar to before training, n-back performance declined with increasing WM load (healthy older adults: F_(3,60)_ = 83.65, *p* < .001, η_p_^2^ = .807; MCI patients: F_(3,36)_ = 39.56, *p* < .001, η_p_^2^ = .767). Training did not affect n-back performance in the healthy older adults. In contrast, MCI patients showed improved performance at 0-back (F_(1,13)_ = 6.82, *p* = .022, η_p_^2^ = .344), and, at trend level, at 1-back (F_(1,13)_ = 4.33, *p* = .058, η_p_^2^ = .250). As a result, after training there was no longer a difference in n-back performance between groups (Table 2).

### Prefrontal activation

#### Before training

**Fig. 2:**
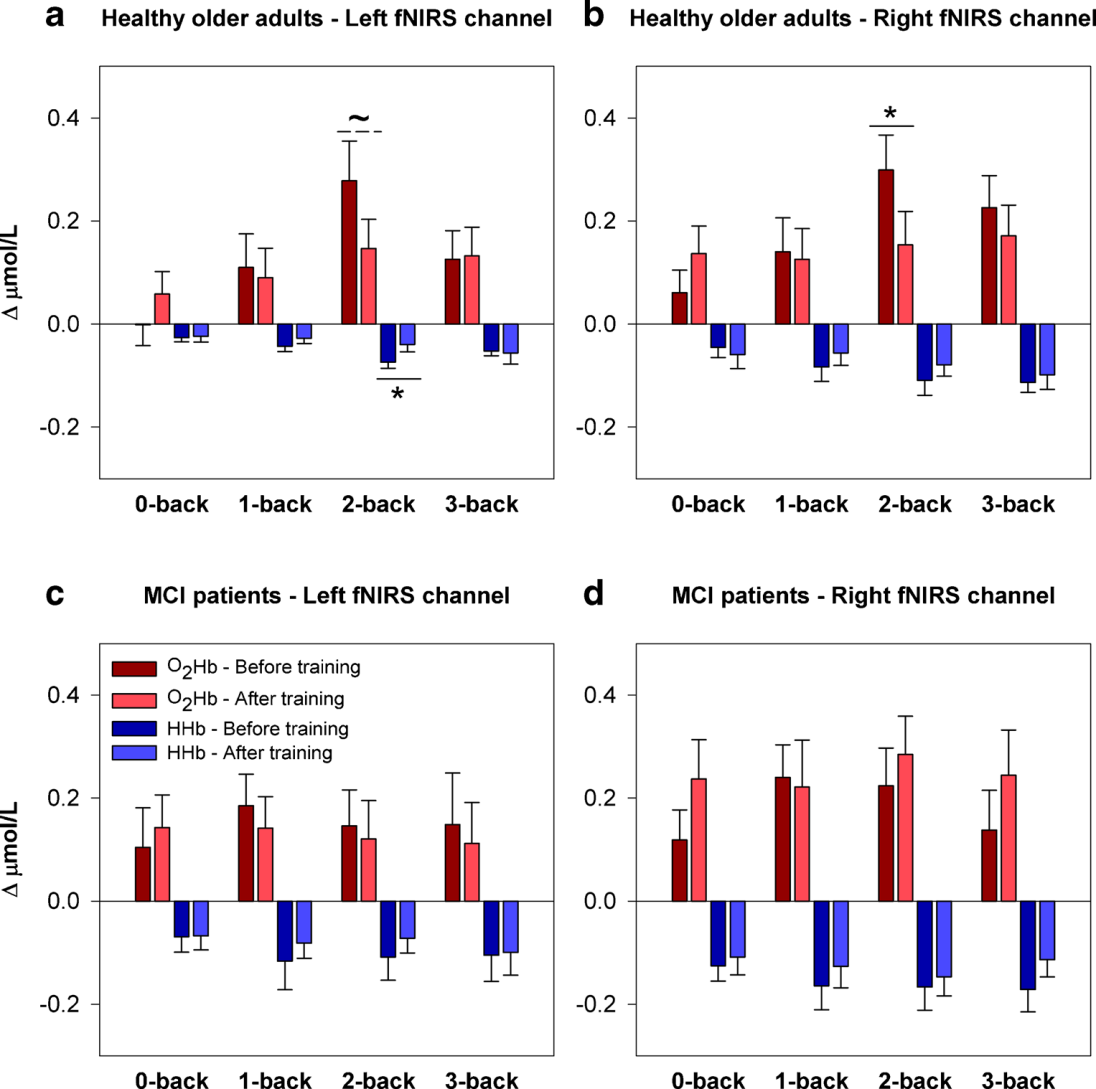
Hemodynamic concentration changes during n-back performance before and after training. Mean ± SEM changes of [O_2_Hb] and [HHb] in the left and right hemisphere in healthy older adults (**a, b**) and MCI patients (**c**, **d**). *p < .05, ^~^ *p* < .10

Figure 2 displays the fNIRS results in both groups. Whole-group analyses revealed main effects of WM load on prefrontal activation at baseline ([O_2_Hb] left: F_(3,99)_ = 2.82, *p* = .043, η_p_^2^ = .079; [O_2_Hb] right: F_(3,99)_ = 5.17, *p* = .002, η_p_^2^ = .136; [HHb] left: F_(3,99)_ = 4.11, *p* = .009, η_p_^2^ = .111; [HHb] right: F_(2.47,81.55)_ = 3.11, *p* = .039, η_p_^2^ = .086). A group effect at trend level was found for [HHb] in the right hemisphere (F_(1,33)_ = 3.48, *p* = .071, η_p_^2^ = .095). Exploratory testing revealed a larger decrease of [HHb] in the right hemisphere in MCI patients compared to healthy older adults during 0-back performance (F_(1,33)_ = 5.44, *p* = .026, η_p_^2^ = .142). No WM load by group interactions were found, although an interaction at trend level was present for [O_2_Hb] in the right hemisphere (F_(3,99)_ = 2.33, *p* = .079, η_p_^2^ = .066). Further testing revealed a significant WM load by group interaction for the 2-back versus 1-back contrast ([O_2_Hb] right: F_(1,33)_ = 4.14, *p* = .050, η_p_^2^ = .111), with an increase of [O_2_Hb] in healthy older adults and no change in MCI (see Fig. 2).

In healthy older adults, WM load effects were present in both left hemisphere (0-back vs. 2-back: [O_2_Hb] *p* = .003, η_p_^2^ = .368, [HHb] *p* = .002, η_p_^2^ = .385; 0-back vs. 3-back: [O_2_Hb] *p* = .048, η_p_^2^ = .181, [HHb] *p* = .032, η_p_^2^ = .211; 1-back vs. 2- back: [O_2_Hb] *p* = .014, η_p_^2^ = .268, [HHb] *p* = .008, η_p_^2^ = .304; 2-back vs. 3-back: [O_2_Hb] *p* = .011, η_p_^2^ = .280, [HHb] trend *p* = .068, η_p_^2^ = .157) and right hemisphere (0-back vs. 2-back: [O_2_Hb] *p* = .001, η_p_^2^ = .418, [HHb] trend *p* = .062, η_p_^2^ = .163; 0-back vs. 3-back: [O_2_Hb] *p* = .017, η_p_^2^ = .254, [HHb] *p* = .030, η_p_^2^ = .214; 1-back vs. 2- back: [O_2_Hb] *p* = .015, η_p_^2^ = .260, [HHb] p = n.s.). MCI patients showed no effect of WM load on the hemodynamic response at baseline.

#### After training

No main effects of WM load and group on prefrontal activation or interaction effects of these factors remained after training. Separate analyses of both groups indicated that in healthy older adults, the [HHb] response was larger in the left hemisphere during 3-back performance compared to 0-back performance (*p* = .033, η_p_^2^ = .209). No other WM load effects were found (Fig. 2).

With respect to training effects, in healthy older adults, an interaction effect of training and WM load was found for [O_2_Hb] (left: F_(1.88,37.62)_ = 2.76, trend level *p* = .079, η_p_^2^ = .121; right: F_(1.93,38.62)_ = 3.73, *p* = .034, η_p_^2^ = .157), but not for [HHb]. Further exploration of this interaction revealed a decrease of prefrontal activation after training specifically and only during 2-back performance (Fig. 2). In the left hemisphere, the [HHb] response (F_(1,20)_ = 4.65, *p* = .043, η_p_^2^ = .189) and the [O_2_Hb] response (F_(1,20)_ = 4.05, trend level *p* = .058, η_p_^2^ = .168) were reduced after training. In the right hemisphere, the [O_2_Hb] response (F_(1,20)_ = 5.24, *p* = .033, η_p_^2^ = .207) showed a reduction, but the [HHb] response did not change. No training effects were found in the MCI group.

### Prediction of training gain

#### Decliners vs. low training gainers vs. high training gainers

Decliners had reduced behavioral performance on the n-back tasks after training (−8.0 ± 4.8 %; t_(9)_ = 3.94, *p* = .003). Low training gainers (+4.7 ± 2.2 %; t_(9)_ = −5.28, *p* = .001) and high training gainers (+18.4 ± 5.8 %; t_(9)_ = −8.11, *p* < .001) had improved behavioral performance. Before training, low gainers performed worse than decliners at the n-back tasks (t_(18)_ = 2.46, *p* = .024). No group differences in n-back performance remained after training, although high gainers showed a tendency towards better performance than low gainers (t_(18)_ = − 1.77, trend *p* = .093).

Before training, high gainers had a stronger [O_2_Hb] response than low gainers in the left hemisphere during 2-back performance (t_(18)_ = −2.59, *p* = .018), as well as a stronger [O_2_Hb] response in the right hemisphere during 3-back performance (t_(18)_ = −3.43, *p* = .003). Similarly, in comparison to decliners, high gainers showed a stronger [O_2_Hb] response in the left hemisphere during 2-back performance (t_(18)_ = −2.29, *p* = .034), and a stronger [O_2_Hb] response in the right hemisphere during 3-back performance (t_(18)_ = −2.33, *p* = .032). Hemodynamic responses of low gainers and decliners did not differ, although low gainers tended to show a stronger [HHb] response in the left hemisphere during 3-back performance (t_(18)_ = 2.07, trend *p* = .053).

In additional analyses, baseline task activation (1-, 2-, 3-back) was corrected for activation in the control condition (0-back). The comparison between low and high gainers did not result in significant differences, although high gainers tended to show a larger increase of [O_2_Hb] in the right hemisphere between the control condition (0-back) and high WM load (3-back) than low gainers (t_(18)_ = −2.07, trend *p* = .054). The difference in hemodynamic response between 0-back and 3-back was larger in high gainers than in decliners in both left hemisphere ([O_2_Hb]: p = n.s.; [HHb]: t_(18)_ = 2.23, *p* = .039) and right hemisphere ([O_2_Hb]: t_(11.37)_ = −2.24, *p* = .046; [HHb]: t_(18)_ = 2.19, *p* = .042). Furthermore, a trend was found for the corrected 2-back task, with a larger [HHb] response in the right hemisphere in high gainers than in decliners (t_(18)_ = 2.07, trend *p* = .053). Finally, in comparison to decliners, low gainers had a stronger [HHb] response in the right hemisphere at corrected 2-back (t_(18)_ = 2.44, *p* = .025) and 3-back (t_(18)_ = 2.13, *p* = .047). They also showed a trend towards an increased [O_2_Hb] response at corrected 1-back (Left: t_(18)_ = −2.04, trend *p* = .056; Right: t_(18)_ = −2.04, trend *p* = .057).

#### Correlations

**Fig. 3:**
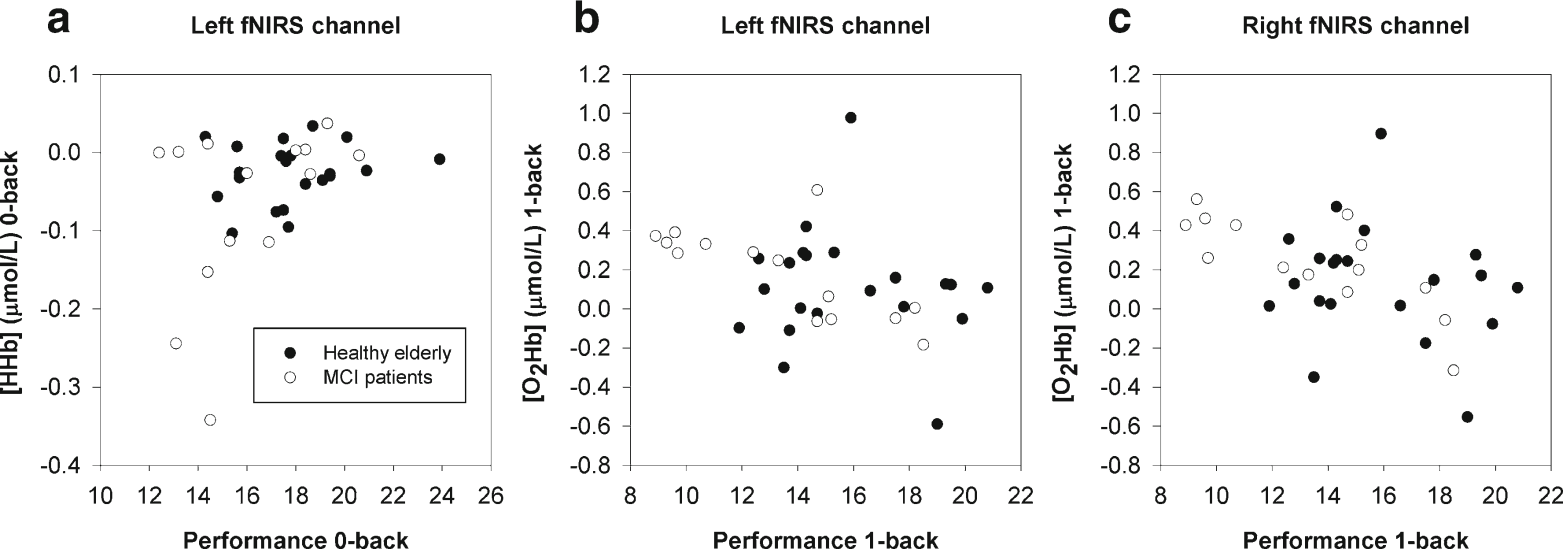
Correlation of hemodynamic response at baseline and behavioral performance (composite A′ score) at baseline. Scatter plots show [HHb] response in left hemisphere at 0-back **a**, and [O_2_Hb] response in left **b** and right hemisphere **c** at 1-back

Correlations were calculated to investigate the relationship between the hemodynamic response at baseline and respectively baseline behavioral performance and training gain in the whole group. A stronger hemodynamic response was associated with worse behavioral performance at 0-back (Fig. 3a, [HHb] Left: *r* = .429, *p* = .013, extreme outlier corrected *r* = .390, *p* = .027) and 1-back (Fig. 3b, [O_2_Hb] Left: *r* = −.396, *p* = .022; Fig. 3c, [O_2_Hb] Right: *r* = −.459, *p* = .007). No such correlations were found for the 2-back and 3-back task.

**Fig. 4:**
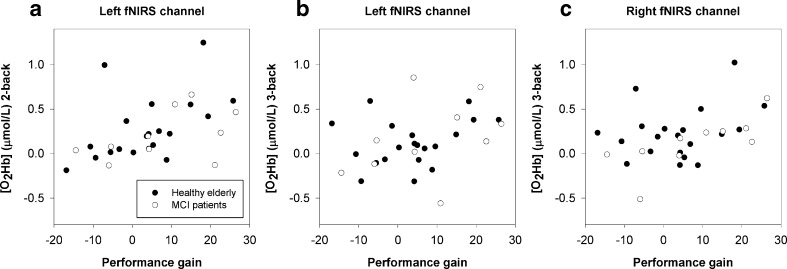
Correlation of hemodynamic response at baseline and behavioral training gain Scatter plots show [O_2_Hb] response in left hemisphere at 2-back **a**, and [O_2_Hb] response in left **b** and right hemisphere **c** at 3-back.

**Fig. 5:**
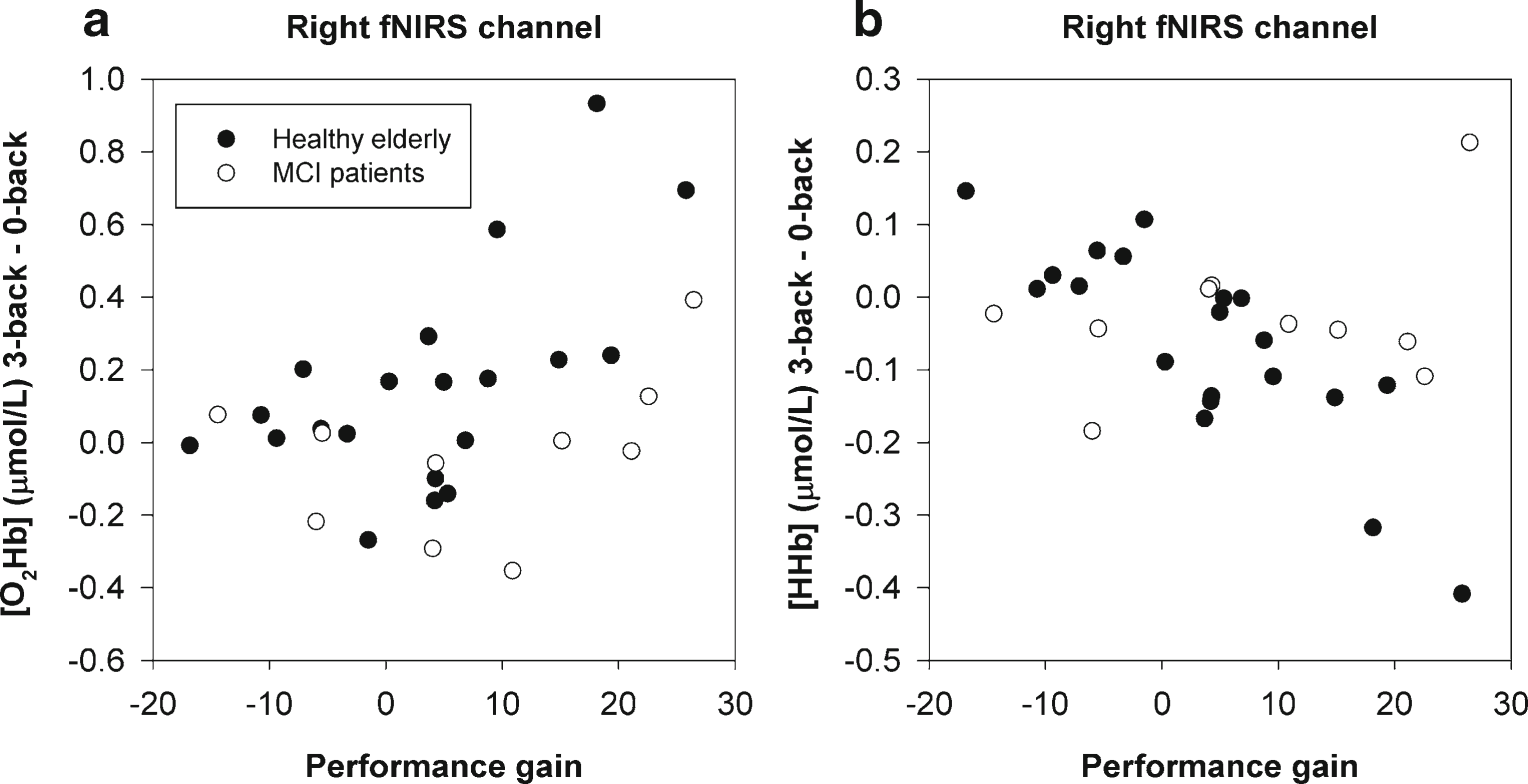
Correlation of hemodynamic response at baseline, corrected for control condition, and behavioral training gain. Scatter plots show [O_2_Hb] response **a** and [HHb] response **b** in right hemisphere for 3-back minus 0-back contrast

Stronger increases of [O_2_Hb] at baseline during performance of the 2-back task (Fig. 4a, Left: *r* = .504, *p* = .006) and 3-back task (Fig. 4b, Left: *r* = .377, *p* = .048; Fig. 4c, Right: *r* = .409, *p* = .031) were associated with a larger training gain. Furthermore, a larger increase in hemodynamic response in the right hemisphere from the control condition (0-back) to high WM load (3-back) was related to a larger training gain (Fig. 5a, [O_2_Hb]: *r* = .387, *p* = .042; Fig. 5b, [HHb]: *r* = −.413, *p* = .029).

### Systemic measurements

Table 3 shows the task-evoked effects on mean arterial pressure in the finger and heart rate in the total group. No group effects were found. Task performance induced a significant increase in mean arterial pressure during pre- and post-training measurements. A task-evoked increase in heart rate was only present after training. Resting heart rate tended to be higher before training (*p* = .059). This may have been due to novelty effects and may provide an explanation for the lack of task-evoked effects on heart rate during pre-training measurements. No other pre- vs. post-training effects were present.

**Table 3:** Mean arterial pressure in the finger and heart rate during 5-min rest measurements and n-back performance

	Mean arterial pressure (mmHg)						Heart rate (beats per minute)					
	Pre-training			Post-training			Pre-training			Post-training		
Rest	76.7	±	12.2	74.7	±	13.1	68.6	±	9.5	66.9	±	8.1
0-back	86.5	±	12.2**	85.5	±	14.3**	70.0	±	8.4^~^	70.7	±	8.1**
1-back	88.5	±	12.6**	86.9	±	14.8**	69.8	±	8.6	70.7	±	8.0**
2-back	90.9	±	14.5**	89.9	±	14.6**	70.5	±	9.5^~^	71.4	±	8.8**
3-back	89.5	±	13.4**	89.9	±	14.5**	69.6	±	9.8	70.1	±	8.2**

Note.**p < .001 *p < .05 ^~^trend .05 < *p* < .10 n-back task performance vs. rest. Physiological data were available from 31 participants (*n* = 19 healthy older adults; *n* = 12 MCI patients)

## Discussion

The aim of our study was to gain more insight into the adaptive responses of the prefrontal cortex in normal aging and MCI. We used fNIRS to compare prefrontal activation during verbal n-back performance at varying levels of WM load between healthy older adults and patients with MCI, and to examine changes in prefrontal activation following 25 sessions of adaptive WM training. Furthermore, we investigated the relationship between WM load-dependent prefrontal activation and behavioral training gain.

### Group differences in prefrontal activation and behavioral performance at baseline

Our first hypothesis was that MCI patients would show stronger prefrontal activation at lower levels of WM load than healthy older adults in order to maintain behavioral performance. The baseline results of the current study partially support this hypothesis. Prefrontal activation was modulated by WM load in healthy older adults, but, in contrast to our expectations, not in MCI. Based on visual inspection of the hemodynamic responses, MCI patients showed maximum increase of [O_2_Hb] at lower WM load (1-back) compared to healthy older adults (2-back). However, examination of the [HHb] responses did not provide a clear view. Behavioral performance and activation of the prefrontal cortex did not differ between groups at high WM load (2-back, 3-back). With respect to low WM load (0-back, 1-back), one group effect was present; MCI patients showed a stronger [HHb] response in the right hemisphere during 0-back performance. In addition, MCI patients performed worse at the low WM load tasks than healthy older adults.

Consistent with previous studies, healthy older adults showed modulation of prefrontal activation in response to verbal WM load (Cappell et al. 2010; Mattay et al. 2006; Nyberg et al. 2009; Prakash et al. 2012; Sala-Llonch et al. 2012; Vermeij et al. 2012). Prefrontal activation increased up to 2-back, with a drop at 3-back, suggesting that the limits of WM capacity were exceeded (Schneider-Garces et al. 2009). This inverted U-shape of the WM load-hemodynamic response curve is in agreement with Heinzel et al. (2014), while, for example, Mattay et al. (2006) found a consistent decrease in activation with increasing load in their n-back study. This variation in the shape of the WM load-hemodynamic response curve may depend on factors such as task difficulty, task design, and population (Stern et al. 2012).

In accordance with the CRUNCH model (Reuter-Lorenz and Cappell 2008), we hypothesized a leftward shift of the WM load-hemodynamic response curve in MCI patients in comparison to healthy older adults. However, no effect of WM load on prefrontal activation was present in MCI. Previous studies on n-back performance suggest responsivity of frontal areas to WM load in MCI (Alichniewicz et al. 2012; Döhnel et al. 2008; Migo et al. 2015). The outcome of studies that directly compared brain activation evoked by a WM challenge between healthy older adults and MCI patients is mixed. Alichniewicz et al. (2012) reported decreased activation in frontal areas in MCI compared to healthy older adults at high WM load, while no group differences occurred at low WM load. Reduced activation in MCI was also found by Saykin et al. (2004). Other studies reported either increased activation in frontal areas in MCI (Bokde et al. 2010; Yetkin et al. 2006), no group differences in brain activation (Döhnel et al. 2008), or group differences in only other than frontal areas such as the anterior cingulate and precuneus (Kochan et al. 2010), as well as the insula, lingual gyrus and hippocampus (Migo et al. 2015). Taken together, the lack of WM load effects in MCI patients in the current study may indicate that the measured prefrontal areas were not responsive to WM load in MCI. However, the absence of group effects may, alternatively, point to limited statistical power due to a small sample of MCI patients or a high variation in hemodynamic responses in the MCI group.

The lack of overall group differences in prefrontal activation at different levels of WM load may suggest that WM processing is unimpaired in MCI. Indeed, high WM load (2-back, 3-back) performance did not differ between groups. However, in comparison to healthy older adults, MCI patients performed worse at low WM load (0-back, 1-back) and they showed a stronger [HHb] response in the right hemisphere at 0-back performance. Previous studies reported unimpaired N-back performance in amnestic MCI patients (Döhnel et al. 2008; Migo et al. 2015), while other studies found lower accuracy at 2-back, but not at 0-back (Alichniewicz et al. 2012), a tendency towards lower accuracy at 2-back (Saykin et al. 2004), or longer reactions times (Rombouts et al. 2005). Of note, in the current study, behavioral performance was assessed by a composite score to take speed/accuracy trade-offs into account and to diminish the influence of strategy effects (McNamara and Scott 2001). This composite measure might be more sensitive to WM impairment. A possible explanation for the baseline results in our study could be that WM abilities are declined in amnestic MCI, posing a challenge already at low WM load, and that only high WM load tasks were challenging for healthy older adults, leading to equal performance in both groups at high WM load. In line with this idea is our finding that, in the whole group, stronger activation at 0-back and 1-back was related to worse performance. Therefore, we propose that increased prefrontal activation at low WM load may reflect inefficient neural processing or unsuccessful (or attempted) compensation.

### Training effects

We hypothesized that adaptive WM training would result in increased processing efficiency in healthy older adults and to restoration of the prefrontal compensatory network in MCI, leading to decreased prefrontal activation in healthy older adults and increased prefrontal activation in MCI patients during performance of a high-demanding WM task. We found decreased bilateral activation in healthy older adults only at 2-back. This group did not show improvement of n-back performance after training. The pattern of decreased activation together with unchanged behavioral performance may be interpreted as improved processing efficiency, since fewer neural resources needed to be recruited in order to meet the task demands (Lövdén et al. 2010). This is in line with several previous studies reporting decreased activation of WM-related frontoparietal areas after process-specific training in healthy older adults (Brehmer et al. 2011; Dahlin et al. 2008; Heinzel et al. 2014), although Erickson et al. (2007) found decreases in activation (left and right dorsolateral prefrontal cortex, right ventrolateral prefrontal cortex) as well as increases (left ventrolateral prefrontal cortex).

Given that a training effect was specifically and only present for the 2-back task, we do not consider it plausible that this would be due to a general test-retest effect, such as changed arousal of the participants. No changes in behavioral performance or activation were found for the 0-back and 1-back task. Possibly, the healthy older adults already performed at an optimal level at baseline, leaving little room for improvement. The drop in pre-training 3-back activation in comparison to 2-back activation suggests a limit in WM capacity. A training effect reflecting improved processing efficiency may only occur at load levels associated with a maximal hemodynamic response, which is 2-back in the current study. In terms of the framework of Lövdén et al. (2010), the necessary mismatch between functional supply and task demands to induce cognitive plasticity may not occur if WM load is far beyond or fully within the current range of cognitive flexibility of the participant.

After training, MCI patients had improved their low WM load performance up to the same level as healthy older adults. In contrast to our expectations and other studies (Rosen et al. 2011; Hampstead et al. 2011, 2012; Belleville et al. 2011), we did not find any training-related effect on brain activation. The patients with MCI who were selected for this study were all of the amnestic subtype. Although there is some evidence that Alzheimer pathology may underlie amnestic MCI (Schneider et al. 2009), we cannot rule out that the pathology underlying our patients’ memory problems may have varied within this group, leading to a ranging ability to show cognitive plasticity. Despite the lack of evidence for training-induced prefrontal activation changes, we previously showed that this WM training had beneficial effects in the current study sample at a behavioral level (Vermeij et al. 2015). Specifically, we found reliably improved performance at WM tasks in both healthy older adults and MCI patients after training, although no generalization to other cognitive domains was present.

### Prediction of training gain

Finally, we hypothesized that prefrontal activation would be predictive of behavioral training gain. Participants who showed a high training gain had a stronger hemodynamic response at high WM load at baseline in comparison to participants who showed a low training gain or declined behavioral performance after training. In line with these results, correlational analyses indicated a positive relationship between high WM load activation at baseline and training gain. These findings held also true when 3-back activation was corrected for 0-back activation. Our study is consistent with previous research showing that a more ‘youth-like’ pattern of activation, that is, a relatively small hemodynamic response at low WM load and a relatively large hemodynamic response at high WM load (Nagel et al. 2009, 2011), is associated with larger behavioral training gain (Heinzel et al. 2014). Thus, it seems that older persons who are able to more strongly recruit prefrontal areas at the high task demand, may demonstrate larger cognitive plasticity, possibly regardless of clinical status.

### Limitations and future directions

This study has some limitations. First, the hemodynamic response was assessed in the prefrontal cortex, but not in other brain areas. We were specifically interested in prefrontal compensatory mechanisms, but we acknowledge that the WM training may have induced activation changes or connectivity changes in a broad network of areas involved in WM processing, including the hippocampus. Second, the sample sizes in the group comparisons were modest, thereby increasing the risk of type II errors. However, sample sizes were comparable to other neuroimaging studies in the field, especially in MCI patients, and the correlational analyses on the full study sample increased the power to find associations between prefrontal activation and behavioral performance. Third, we did not include a non-trained control group in this study. However, the intention of this study was to establish the neural correlates of WM training, but not to determine the efficacy of WM training as a clinical intervention. This study may therefore provide some leads for a randomized controlled trial with a larger sample of patients.

Future research should further examine individual differences in prefrontal compensatory recruitment and cognitive training outcome, and needs to identity factors that may limit or stimulate cognitive plasticity in older adults. This also emphasizes the need for longitudinal studies to be able to detect subtle within-person changes in activation over time (Kennedy et al. 2015) and to enable stratification of MCI patients based on later-life diagnoses such as Alzheimer’s dementia.

Finally, by including systemic measurements in the current study, we showed task-evoked increases in mean arterial pressure and heart rate. Whereas cerebral blood flow is largely stabilized by cerebral autoregulation, skin blood flow is more strongly affected by systemic fluctuations. Task-evoked changes in skin perfusion may obscure changes in cerebral activation in fNIRS studies (Kirilina et al. 2012; Tachtsidis et al. 2009; Vermeij et al. 2014). Further studies are needed to better characterize conditions under which systemic artifacts occur in the fNIRS signal, and to develop methods to separate the extracranial and cerebral contributions to the fNIRS signal.

## Conclusions

In conclusion, we found evidence for increased prefrontal processing efficiency in healthy older adults after WM process-specific training. This effect occurred at a task that presumably posed maximal challenge within the cognitive flexibility range. Although MCI patients had improved behavioral performance at low WM tasks after training, we did not find evidence for training-induced changes in prefrontal activation in this group. In line with the CRUNCH model (Reuter-Lorenz and Cappell 2008), a ‘youth-like’ pattern of prefrontal activation at older age (also in MCI) seems to be beneficial for behavioral performance and behavioral training gain; relatively stronger prefrontal recruitment at high WM load was related to higher gain. This may imply that older persons with a ‘youth-like’ brain response might demonstrate larger cognitive plasticity. In contrast, relatively stronger prefrontal recruitment at low WM load was related to worse behavioral outcome. Hence, these activation increases may be attributed to inefficient processing or unsuccessful prefrontal compensation.
